# Influence of Different Cooking Methods on Fillet Steak Physicochemical Characteristics

**DOI:** 10.3390/ijerph19010606

**Published:** 2022-01-05

**Authors:** Vinícius Lopes Borela, Ernandes Rodrigues de Alencar, Marcio Antônio Mendonça, Heesup Han, António Raposo, Antonio Ariza-Montes, Luis Araya-Castillo, Renata Puppin Zandonadi

**Affiliations:** 1 Department of Nutrition, Faculty of Health Sciences, Campus Darcy Ribeiro, University of Brasilia (UnB), Asa Norte, Brasilia 70910-900, Brazil; viniborela@gmail.com (V.L.B.); renatapz@yahoo.com.br (R.P.Z.); 2 Faculty of Agronomy and Veterinary, Campus Darcy Ribeiro, University of Brasilia (UnB), Asa Norte, Brasilia 70910-900, Brazil; ernandesalencar@gmail.com (E.R.d.A.); marcioamen@gmail.com (M.A.M.); 3 College of Hospitality and Tourism Management, Sejong University, 98 Gunja-Dong, Gwanjin-Gu, Seoul 143-747, Korea; 4 CBIOS (Research Center for Biosciences and Health Technologies), Campo Grande 376, Universidade Lusófona de Humanidades e Tecnologias, 1749-024 Lisboa, Portugal; 5 Social Matters Research Group, Universidad Loyola Andalucía, C/Escritor Castilla Aguayo, 4, 14004 Cordoba, Spain; ariza@uloyola.es; 6 Facultad de Administración y Negocios, Universidad Autónoma de Chile, Santiago 7500912, Chile; 7 Facultad de Economía y Negocios, Universidad Andrés Bello, Santiago 7591538, Chile; luis.araya@unab.cl

**Keywords:** cooking methods, steak fillet, air fryer, pan fryer, oil

## Abstract

Meat is a source of protein widely consumed by the population in many countries due mainly to the nutritional aspects, sensory characteristics, and cultural aspects. The meat cooking preparation can promote significant changes in the meat’s chemical composition and physical characteristics. Such transformations can impact both the acceptance of the product and consumers’ health. Due to the different thermal processes altering the physical-chemical characteristics of meat, the present study aimed to evaluate the physicochemical characteristics of fillet steak submitted to different cooking methods: pan-frying with and without oil and air fryer. We performed the analysis to evaluate the physicochemical characteristics considering moisture, lipid, protein, ash, sodium, and potassium content, cooking loss index and colorimetry in three degrees of doneness of the meat, rare, medium, and well done. The fillet steak prepared by pan-frying with oil lost higher moisture and weight than the other samples. The air fryer method presented the highest moisture content. There was a significant difference in lipid content in which the pan-frying with oil fillet steak showed the highest amount of lipids. The pan-frying with oil steak fillet also presented more changes in the colorimetric parameters evaluated compared to the other samples. The pan-frying with oil cooking method promoted more pronounced changes in the steak fillet, and the cooking air fryer, the changes in meat quality are less pronounced. Therefore, the air fryer can be considered a good alternative for cooking meat.

## 1. Introduction

Meat is a source of protein widely consumed by the population in many countries due mainly to the nutritional aspects (the content of proteins of high biological value, iron, and vitamin B12 content) as a strategy to avoid malnutrition in some populations, and its sensory characteristics and the cultural aspects related to the meat consumption (varying according to demographic aspects, such as age, gender, employment and education status and others) [1,2,3]. Since ancient times, meat has been an important component of the human diet. Meat consumption is linked to the idea that a healthy diet should include meat (to achieve adequate ingestion of protein and iron), and to masculinity justifying the higher ingestion of meat by males compared to females [3,4,5]. Even though the type and amount of meat ingested differ among populations and cultures, most Western countries’ dishes include meat accompanied by vegetables [1]. Almost a half of the world’s meat production is from European countries, Brazil and the United States. The global per capita meat consumption is estimated at around 35 kg per year [6]. Meat consumption highly increased in the last 20 years, and the trend seems to continue [7,8]. With the increase in the global population, it is estimated to rise steadily in overall meat consumption. However, people are concerned with the impact of meat consumption (mainly red meat) on health and the environment [6,9].

Despite its nutritional contribution to humans, some studies have demonstrated that the large quantity, the high frequency of consumption, and the meats’ preparation can contribute to the development of chronic diseases like cardiovascular diseases, dyslipidemias, cancer, and diabetes [10,11,12,13,14,15]. Nevertheless, in 2019, the Annals of Internal Medicine published a dietary guideline recommendation claiming that there is no need to reduce red meat for good health [16], but it is necessary to evaluate the meat preparation to avoid health damages. The influence of different cooking methods on the physicochemical properties of foods is a topic that concerns scientists, cooks, chefs and consumers [17]. Cooking is a heating operation frequently applied to meat before consumption. Cooking coagulates and denatures meat proteins, improves palatability, reduces the number of microorganisms, improves the storage life of meat products, inactivates proteolytic enzymes, and modifies the texture or tenderness of meat and meat products [18]. The meat cooking preparation can promote significant changes in the meat’s chemical composition and physical characteristics. Such transformations can impact both the acceptance of the product and consumers’ health. Different meat preparation methods impact the sensory quality of the meat and its acceptability since they can promote dimming, hardening, or loss of tenderness, loss of taste, and odor [18]. Some cancer studies have found an increased risk with increased red meat cooking time and temperature [15,19,20,21]. Therefore, meat preparation techniques have been explored to promote food consumption with less negative health impacts, linked to these foods’ chemical composition after preparation [18].

Due to the different thermal processes altering the physical-chemical characteristics of meat, it is important to analyze the changes in meat using different cooking methods. Additionally, to the best of our knowledge, there is no study about the quality of the meat prepared on an air fryer (a method that employs rapid hot air circulation to “fry” food without oil instead of using hot oil, as traditional frying methods air fryers [22]). Therefore, this study aimed to evaluate fillet steak’s physicochemical characteristics submitted to different cooking methods: pan-frying with and without oil and air fryer and their potential impact on health.

## 2. Materials and Methods

A quantitative experimental study was performed into three stages: (i) sample preparation; (ii) analysis of physicochemical characteristics; (iii) statistical analysis of the data.

### 2.1. Sample Preparation

**Figure 1:**
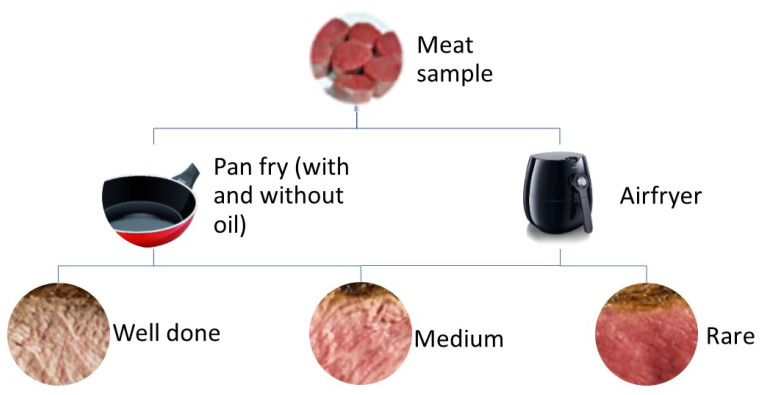
Cooking method schematic.

The refrigerated raw fillet steak was obtained in the commercial establishment of the Federal District, Brazil. The pieces of filet mignon were cut into steaks 2 cm thick [23] and submitted to three different cooking methods (pan-frying with and without oil, and air fryer) in three degrees of doneness of the meat, rare, medium and well done. For the doneness of the meat, the internal temperatures of the geometric center of the meat were considered: 60 °C (rare), 70 °C (medium) and 75 °C (well done) [24] (Figure 1).

We used a non-stick frying pan (Polishop^®^) and a conventional gas stove as a source of heat for pan-frying with and without oil. We preheated 100 g of soybean oil (180 °C) for pan-frying cooking with oil before the meat sample’s insertion. The oil was renewed at each preparation to avoid affecting the amount of oil absorbed by the fillets [25]. For air fryer cooking, we used the electric air Fryer Philips^®^ preheated for 5 min (reaching the temperature of 200 °C) before inserting the fillet steak sample, as recommended by the manufacturer. 

We standardized the time (min) necessary to reach the temperatures of the geometric center of each meat doneness as described by Wright and Treuille [24] (Table 1). 

### 2.2. Analysis of Physicochemical Characteristics

For each analysis, we used three samples of steak fillet for each treatment (3 cooking methods × 3 meat doneness × 3 units = 27 samples). The Physicochemical analysis was performed in triplicate. 

#### 2.2.1. Chemical Analysis

According to the Adolfo Lutz Institute, we determined the moisture content using the gravimetric method (oven at 105 °C for 24 h) [26]. We used the AOAC method [27] to quantify protein (method 920.152), and ash (method 942.05). We performed lipid content analysis using petroleum ether extraction using the Ankom Extraction System (Model ANKOM XT10 Extractor, ANKOM Technology, Macedon, NY, USA) by the Am 5-04 method [28]. This method is used to determine lipid in meat, feed and other foods and is equivalent to oil extraction performed by the Soxhlet extraction apparatus [28]. It is important to mention that the oil extraction takes place in the sealed vessel with solvent at 90–100 °C, which implies an acceleration of the extraction kinetics, with a maximum extraction time of 60 min [29]. The sodium and potassium contents determination was carried out in flame photometer AP-1302, according to the method 969.23 [27]. The equipment was previously calibrated using standard solutions of the analyzed minerals (Na and K), according to the concentrations established for the equipment and supplied by the manufacturer.

#### 2.2.2. Cooking Factor Index

The cooking factor index was obtained from the relation between the weight of the cooked meat (WCM) (in grams) and the weight of the raw meat (WRM) (in grams) [30]. In this sense, the lower the cooking factor index, the higher the cooking loss [30].

Cooking factor index = WCM/WRM(1)

#### 2.2.3. Colorimetry

Meat color evaluation was performed using the ColorQuest^XE^ Spectrophotometer (HunterLab, Reston, VA, USA). The color of each meat sample’s surface and center was analyzed, obtaining the values of the coordinates L, a, and b of the Hunter system. From L, a and b values, it was possible to obtain parameters related to the color difference ΔE (Equation (2)), hue angle h (Equation (3)) and color saturation or chroma C (Equation (4)) [31,32,33,34].

(2) $$\Delta E=\sqrt{{\left(L-L_{0}\right)}^{2}+{\left(a-a_{0}\right)}^{2}+{\left(b-b_{0}\right)}^{2}}$$

h = arctang (b/a)(3)

(4) $$C=\sqrt{\left(a^{2}+{\;b}^{2}\right)}$$

where:

L = measurable in terms of white to black intensity; a = measurable in terms of red and green intensity; b = measurable in terms of yellow and blue intensity; and L_0_, a_0_, and b_0_ refer to raw meat.

### 2.3. Statistical Analysis

A completely randomized design was used in the 3 × 3 Factorial Scheme, with three cooking methods and three meat doneness, with three replicates. Analysis of variance was performed to verify, initially, whether the interaction between cooking methods and meat doneness was significant (*p* < 0.05). When this interaction was not significant (*p* > 0.05), the factors’ effect alone was verified. Tukey’s test at 5% probability was adopted for the average test. StatPlus v.5 software (AnalystSoftInc., Vancouver, BC, Canada) was used for statistical analysis.

## 3. Results

Table 2 shows the chemical composition of the raw and cooked fillet steak samples (100 g) and the samples’ cooking loss factor index after the heat treatment. There was a significant variation (*p* < 0.05) due to the interaction between cooking methods and meat doneness for moisture content, protein content, lipid content, ash content, potassium content and cooking factor index (Table 2). On the other hand, there was no significant variation (*p* > 0.05) for sodium content due to the interaction between cooking methods and meat doneness, and to these factors alone.

As expected, the moisture content was the lowest value for well done meat doneness in all cooking methods. In all cooking methods, the moisture content was significantly reduced (*p* < 0.05) with the increasing exposure of the meat to heat. It is important to highlight that when comparing the different cooking methods for well-done meat doneness, the lowest mean value obtained was 54.27 ± 0.27 g/100 g (pan-frying with oil), while the highest value was 65.11 ± 0.39 g/100 g (air fryer). There was a significant difference (*p* < 0.05) regarding the protein content, comparing the mean values of meat doneness in the different cooking methods. The highest protein contents were observed for well-done meat doneness, with an average value of 34.19 ± 0.90 g/100 g obtained when using pan-frying with oil. There was no significant difference in lipid content (*p* > 0.05) comparing the mean values obtained in the different meat doneness for pan-frying without the oil method. For the air fryer method, there was a significant difference (*p* < 0.05) when comparing the mean value obtained in rare meat doneness (2.63 ± 0.45 g/100 g) and well-done meat doneness (4.59 ± 0.13 g/100 g). As expected, the highest values of lipid content were verified for the different meat doneness when the pan-frying with oil method was adopted. Regarding ash content, the mean value obtained for well done meat doneness (2.48 ± 0.30 g/100 g) was higher (*p* < 0.05) than for rare meat doneness (1.08 ± 0.39 g/100 g) and medium meat doneness (1.31 ± 0.06 g/100 g), when pan-frying without oil was adopted.

For potassium content, the highest values were obtained in pan-frying with the oil method (medium and well-done meat doneness) compared to pan-frying without oil and air fryer. The cooking factor indexes were lower (*p* < 0.05) for all pan-frying samples with oil than those without oil and air fryer samples. The cooking factor index was lower for well-done samples than the other samples, regardless of the cooking method.

The colorimetric analysis of the cooked meat on the surface and the geometric center is presented in Table 3. There was a significant variation (*p* < 0.05) due to the interaction between cooking methods and meat doneness for hue angle (h°), color saturation (C) and color difference (ΔE) on the surface and the geometric center of the samples.

There was a significant difference in hue angle (*p* < 0.05) when comparing the mean meat doneness values in the different cooking methods. The highest mean values were obtained in the center of the samples for well-done meat doneness. When comparing the hue angle in the center of the samples to well-done meat doneness, the lowest mean value was obtained using the air fryer method (63.40 ± 2.99). Regarding the hue angle on the samples’ surface, there was no significant variation when comparing the mean values obtained in the different meat doneness for panfrying without oil and air fryer methods. On the other hand, for pan-frying with the oil method, the lowest value obtained was 44.99 ± 0.93 in well-done meat doneness, compared to rare meat doneness (53.95 ± 1.90) and medium meat doneness (51.90 ± 2.78).

There was a variation in color saturation when comparing the meat doneness among different cooking methods, in the center and on the surface of the samples. In the center of the samples, the lowest color saturation values were obtained for well-done meat doneness. For well-done meat doneness, a higher color saturation value was obtained in the center of the samples when the panfrying without oil method was adopted (8.15 ± 0.62), compared to pan-frying with the oil method (5.39 ± 0.53). On the surface of the samples, there is a significant difference (*p* < 0.05) observed when comparing the mean values obtained for well-done meat doneness between the pan-frying with oil (3.70 ± 0.23) and pan-frying without oil (6.25 ± 0.77) methods and air fryer (6.78 ± 0.38).

The color difference was influenced by cooking time in the different cooking methods (in the center and on the samples’ surface). In the center of the samples, the significant difference observed when comparing the mean values obtained between the different cooking methods for rare meat doneness was highlighted. A mean color difference value of 15.71 ± 1.68 was obtained when the pan-frying with oil method was adopted, while panfrying without oil and air fryer methods, the mean values were equal to 11.69 ± 1.21 and 11.46 ± 0.30, respectively. On the surface of the samples, the significant difference for well-done meat doneness was highlighted. The highest mean value (*p* < 0.05) of color difference on the surface of the samples was obtained when the pan-frying with oil method was adopted (18.70 ± 0.88). On the other hand, the lowest mean values were observed for pan-frying without oil (11.01 ± 1.95) and air fryer (12.69 ± 1.07) methods.

## 4. Discussion

The cooking of meats results in a better aroma and tenderness compared to raw meat. Additionally, cooking meat tends to be more attractive and more digestible while almost sterile [35]. However, heat treatment affects the nutritional quality of meat due to changes in some components. Therefore, the healthy perception of people about meat is also related to its harmful association with their nutritional quality, and other unhealthy contaminants produced or activated during the heat process, such as heterocyclic aromatic amines [36,37,38], since meat consumption has been associated with chronic illnesses, such as obesity, cancer, type-2 diabetes, and cardiovascular-related diseases [11,36,37,38,39]. 

The temperature and duration of cooking tend to considerable influence on the lipid content [40,41] because it can be influenced by the reduction of the moisture as well as the use of a lipid source in the preparation of the dish [30]. In our study, the lipid content was higher in all pan-frying samples with oil than the pan-frying without oil and air fryer samples since we use oil only in the pan-frying with oil meat samples. The meat doneness also influenced the lipid content lower to steak fillet’s rare doneness than the other samples in all cooking methods, probably due to the concentration of nutrients because of the water evaporation during cooking. During the heating process for fillet steaks, cooking losses increase gradually (measured by cooking factor index that decreased gradually) with increased internal temperatures (from rare to well-done doneness), as found by other authors [42]. The loss probably results from protein denaturation completed at temperatures higher than 75 °C [42], and the water evaporation is confirmed by reducing the moisture content among meat doneness (Table 2). The importance of cooking loss (measured by cooking factor) is widely known, contributing to the selection of temperature/time for a cooking process. It is known that cooking loss depends on the kinetic of the mass transfer process during cooking; therefore, different cooking techniques will determine differences in water loss [17]. In our study, there were no significant differences in the cooking factor index (which evaluates the cooking loss) comparing the pan-frying with oil and air fryer samples. However, the pan-frying with oil method had a higher cooking loss (lower cooking factor index–Table 2) than pan-frying samples.

Studies have demonstrated that lipid content can influence heterocyclic amines [40,41]. Therefore, the samples that present high amounts of lipids tend to produce more heterocyclic amines, being less healthy than the other samples. It is important to highlight that the impact of lipid content is lower than the temperature and duration of cooking on heterocyclic amines formation [40,41]. Considering this aspect, the rare doneness meat samples tend to be healthier than the other doneness (except for the pan-frying without oil samples), being the pan-frying with oil method the less healthy considering the possible formation of heterocyclic amines [40,41].

The formation of heterocyclic amines might originate from Maillard reactions between hexoses and free amino acids (mainly creatinine and creatine); however, the exact mechanism has not been established yet [37]. Heterocyclic amines and cooking meat methods at a high temperature or until well-done have often been suggested as risk factors for cancer [38] and other disorders [13,14,39]. It is also important to highlight that, despite the higher content of protein among well-done samples compared to the rare ones (mainly due to protein concentration caused by the water loss that occurred to the higher meat cooking time and temperature), as the temperature increases, a protein starts to unfold [43]. When almost all the tertiary and secondary structures are lost, the unfolded protein may aggregate, have its disulfide bonds scrambled, undergo side-chain modifications, and cross-link with other polypeptides. Heat-induced alteration in the 3-dimensional structure of meat proteins has been shown to cause many quality changes in meat, including color, tenderness, and gelation [43]. However, the quality of a dietary protein’s structure influences its nutritional value and functionality upon gastrointestinal digestion and overcooked meat contained significantly higher protein carbonyl content than those of moderately cooked meat. Unfortunately, the dietary intake of some modified proteins can potentially contribute to the pathogenesis of degenerative diseases, such as diabetes, hepatic and renal fibrosis [43] and further studies should be conducted to evaluate it since it was not the focus of this study.

Meat cooked to the various endpoint temperatures has different characteristics. The color change of meat as it is heated is used to indicate the doneness degree. In our study, the internal temperatures of the geometric center of the meat were considered doneness of the meat: 60 °C (rare), 70 °C (medium) and 75 °C (well done) [24]. The sensory attributes of meat play a central role in consumers’ acceptance [44], and the color is considered one of the sensory aspects important to meat acceptance. More pronounced differences in the meat color occurred on the well-done doneness, as we expected since the meat becomes less red along with the heat exposure, confirmed by the increase of hue angle (Table 3). It occurs since myoglobin is stable to heat up to about 60 °C but precipitates or coprecipitates with other proteins at higher temperatures [42]. In this sense, the meat’s rare doneness tends to be redder in the center than the others (Table 3). When a muscle is heated, fibers shrink, and sarcomeres shorten, resulting in less water-binding ability, and cooking losses occur [18,42]. These muscle fibers changes appear to occur at higher than 70 °C internal temperatures. In this sense, the browner color in meat tends to be less healthy than the red one.

It is also important to highlight the effect of different cooking methods on meat color. More significant changes were observed using a pan-frying with oil steak fillet in which we observed lower color saturation values and higher color difference values than other methods. According to the American Meat Science Association (AMSA) [34], color saturation is the strength of color and the quality by which strong and weak colors are differentiated. Thus, lower saturation color values indicate weaker or less intense colors. On the other hand, higher values of color difference indicate more significant color changes than raw meat, which was used as the standard to analyze this variable [34].

Meat cooking practices and consumption potentially influence health outcomes. However, a potential limitation of our study is the absence of the survey of physiological effects in humans intaking meat with different types of meat cooking methods. Additionally, future research evaluating other meat preparation methods of meat preparation, maturation methods for meat, use of herbs and spices, and use of healthier oils (such as olive oil) might be performed to determine the best meat preparation method considering sensory and physicochemical characteristics and the effects on human health. 

## 5. Conclusions

Different cooking methods (pan-frying with or without oil and air fryer) result in specific physicochemical characteristics in steak fillet. The air fryer seems similar to the pan-frying without oil method regarding the nutritional and physical aspects. The air fryer method and pan-frying without oil last a similar time to cooking, higher than the pan-fryer with oil cooking time. Air fryer and pan-frying without oil methods seem to be healthier than pan-fryer with oil, as well as the rare doneness seem to be better than well-done considering the parameters evaluated. The pan-frying with oil steak fillet also presented more changes in the colorimetric parameters evaluated compared to the other samples. The pan-frying with oil cooking method promoted more pronounced changes in the steak fillet, and the cooking air fryer, the changes in meat quality are less pronounced. Our results can guide actors in food production and consumers to choose different cooking methods influencing fillet steak’s nutritional and physicochemical attributes. Further studies are necessary to compare the other sensory aspects (such as texture and flavor) of the different cooking methods used in meat preparation. 

## Figures and Tables

**Table 1:** Time used for each meat doneness in the different cooking methods.

Meat Doneness	Cooking Method		
	Pan-Frying with Oil	Pan-Frying without Oil	Air Fryer
Rare	1.3 min	2.1 min	2.5 min
Medium	2.0 min	3.2 min	3.0 min
Well done	3.0 min	3.0 min (high fire) + 3.0 min (low fire)	4.1 min

**Table 2:** Chemical composition of raw and cooked fillet steak (pan-frying with oil, pan-frying without oil, and air fryer) and cooking factor index.

Meat Doneness	Cooking Method			
	None	Pan-Frying without Oil	Air Fryer	Pan-Frying with Oil
	Moisture (g/100 g)			
Raw	72.13 ± 0.92			
Rare		69.45 ± 0.47 ^Aa^	70.49 ± 0.24 ^Aa^	62.83 ± 0.57 ^Ab^
Medium		66.38 ± 0.11 ^Bb^	69.06 ± 0.44 ^Ba^	59.49 ± 0.43 ^Bc^
Well done		61.77 ± 0.41 ^Cb^	65.11 ± 0.39 ^Ca^	54.27 ± 0.27 ^Cc^
	Protein (g/100 g)			
Raw	22.90 ± 0.42			
Rare		26.28 ± 0.82 ^Bab^	24.38 ± 0.25 ^Bb^	27.24 ± 1.24 ^Ba^
Medium		28.51 ± 0.29 ^Aa^	25.38 ± 0.46 ^Bb^	28.33 ± 0.36 ^Ba^
Well done		29.67 ± 0.61 ^Ab^	28.19 ± 0.39 ^Ab^	34.19 ± 0.90 ^Aa^
	Lipids (g/100 g)			
Raw	3.10 ± 0.41			
Rare		2.90 ± 0.66 ^Ab^	2.63 ± 0.45 ^Bb^	6.97 ± 0.55 ^Ba^
Medium		3.58 ± 0.23 ^Ab^	3.65 ± 0.37 ^ABb^	9.51 ± 1.02 ^Aa^
Well done		3.83 ± 0.22 ^Ab^	4.59 ± 0.13 ^Ab^	9.76 ± 1.22 ^Aa^
	Ash (g/100 g)			
Raw	1.32 ± 0.05			
Rare		1.08 ± 0.39 ^Ba^	1.29 ± 0.08 ^Aa^	1.66 ± 0.01 ^Aa^
Medium		1.31 ± 0.06 ^Ba^	1.58 ± 0.01 ^Aa^	1.66 ± 0.29 ^Aa^
Well done		2.48 ± 0.30 ^Aa^	1.56 ± 0.23 ^Aa^	2.33 ± 0.35 ^Aa^
	Sodium (mg/100 g)			
Raw	98.09 ± 10.11			
Rare		82.55 ± 1.61 ^Aa^	88.41 ± 4.44 ^Aa^	88.75 ± 4.69 ^Aa^
Medium		75.11 ± 5.90 ^Aa^	82.26 ± 10.04 ^Aa^	94.20 ± 10.24 ^Aa^
Well done		81.22 ± 8.83 ^Aa^	81.83 ± 1.41 ^Aa^	95.90 ± 9.93 ^Aa^
	Potassium (mg/100 g)			
Raw	409.93 ± 11.10			
Rare		468.71 ± 22.47 ^ABa^	488.42 ± 22.28 ^Aa^	455.68 ± 25.40 ^Ba^
Medium		447.78 ± 8.86 ^Bb^	467.76 ± 10.41 ^Aab^	501.72 ± 25.86 ^ABa^
Well done		499.45 ± 23.93 ^Aa^	497.55 ± 9.90 ^Aa^	531.15 ± 32.04 ^Aa^
	Cooking factor index			
Rare	-	0.88 ± 0.01 ^Aa^	0.86 ± 0.01 ^Aa^	0.79 ± 0.02 ^Ab^
Medium		0.83 ± 0.02 ^Aa^	0.83 ± 0.01 ^Aa^	0.70 ± 0.04 ^Bb^
Well done		0.71 ± 0.01 ^Ba^	0.72 ± 0.02 ^Ba^	0.60 ± 0.04 ^Cb^

Means followed by the same capital letters in a column and lower-case letters on the lines do not differ significantly by the Tukey test at 5% of significance.

**Table 3:** Colorimetric characteristics of the cooked steak fillet on the surface and the geometric center.

	Cooking Methods					
	Panfrying without Oil	Air Fryer	Pan Frying with Oil	Pan Frying without Oil	Air Fryer	Pan Frying with Oil
	Center			Surface		
Meat doneness	Hue angle (h°)					
Rare	54.75 ± 3.10 ^Ba^	51.49 ± 3.29 ^Ba^	50.38 ± 3.89 ^Ca^	52.63 ± 3.96 ^Aa^	51.61 ± 3.09 ^Aa^	53.95 ± 1.90 ^Aa^
Medium	59.57 ± 0.98 ^Ba^	55.08 ± 2.06 ^ABa^	60.52 ± 3.78 ^Ba^	54.69 ± 3.36 ^Aa^	54.76 ± 2.59 ^Aa^	51.90 ± 2.78 ^Aa^
Well done	70.61 ± 1.68 ^Aa^	63.40 ± 2.99 ^Ab^	72.07 ± 3.38 ^Aa^	56.60 ± 3.27 ^Aa^	56.11 ± 3.46 ^Aa^	44.99 ± 0.93 ^Bb^
	Color saturation (C)					
Rare	11.00 ± 0.93 ^Aa^	9.97 ± 0.98 ^Aa^	9.05 ± 2.09 ^Aa^	8.25 ± 1.26 ^Aa^	7.61 ± 1.61 ^Aa^	5.64 ± 0.33 ^Aa^
Medium	10.13 ± 0.35 ^Aa^	10.84 ± 0.69 ^Aa^	8.51 ± 1.19 ^Ab^	7.31 ± 0.80 ^Aa^	7.78 ± 1.12 ^Aa^	5.63 ± 1.29 ^Aa^
Well done	8.15 ± 0.62 ^Ba^	6.81 ± 1.67 ^Bab^	5.39 ± 0.53 ^Bb^	6.25 ± 0.77 ^Aa^	6.78 ± 0.38 ^Aa^	3.70 ± 0.23 ^Ab^
	Color difference (ΔE)					
Rare	11.69 ± 1.21 ^Bb^	11.46 ± 0.30 ^Bb^	15.71 ± 1.68 ^Ba^	10.72 ± 2.93 ^Aa^	9.36 ± 1.40 ^Ba^	12.20 ± 2.11 ^Ba^
Medium	14.42 ± 0.93 ^Bb^	17.17 ± 2.22 ^Aab^	19.80 ± 1.61 ^Aa^	11.38 ± 1.55 ^Aab^	9.69 ± 1.07 ^Bb^	13.93 ± 0.54 ^Ba^
Well done	18.34 ± 2.28 ^Aa^	17.17 ± 2.22 ^Aa^	19.80 ± 1.61 ^Aa^	11.01 ± 1.95 ^Ab^	12.69 ± 1.07 ^Ab^	18.70 ± 0.88 ^Aa^

Means followed by the same capital letters in a column and lower-case letters on the lines do not differ significantly by the Tukey test at 5% of significance.

## Data Availability

The study did not report any data.
